# Estimation of the Differential Pathlength Factor for Human Skin Using Monte Carlo Simulations

**DOI:** 10.3390/diagnostics13020309

**Published:** 2023-01-13

**Authors:** Murad Althobaiti

**Affiliations:** Biomedical Engineering Department, College of Engineering, Imam Abdulrahman Bin Faisal University, Dammam 31441, Saudi Arabia; mmalthobaiti@iau.edu.sa

**Keywords:** near-infrared, differential pathlength factor, Monte Carlo, human skin

## Abstract

Near-infrared technology is an emerging non-invasive technique utilized for various medical applications. Recently, there have been many attempts to utilize NIR technology for the continues monitoring of blood glucose levels through the skin. Different approaches and designs have been proposed for non-invasive blood glucose measurements. Light photons penetrating the skin can undergo multiple scattering events, and the actual optical pathlength becomes larger than the source-to-detector separation (optode spacing) in the reflection-mode configuration. Thus, the differential pathlength factor (DPF) must be incorporated into the modified Beer–Lambert law. The accurate estimation of the DPF values will lead to an accurate quantification of the physiological variations within the tissue. In this work, the aim was to systematically estimate the DPF for human skin for a range of source-to-detector separations and wavelengths. The Monte Carlo (MC) method was utilized to mimic the different layers of human skin with different optical properties and blood and water volume fractions. This work could help improve the accuracy of the near-infrared technique in the measurement of physiological variations within skin tissue.

## 1. Introduction

Near-infrared (NIR) is an emerging non-invasive technique utilized for various medical applications. In the past two decades, NIR has been heavily investigated in the applications of breast and brain imaging. For breast imaging, NIR has been used to non-invasively detect breast tumors [1,2,3,4,5,6]. Moreover, NIR was successfully used to non-invasively differentiate between benign and malignant tumors [7,8]. Recently, comparisons have been made with optical systems and traditional breast imaging modalities [4,5,6]. To accurately localize breast lesions, NIR is normally combined with other medical imaging modalities such as ultrasound, X-ray, or magnetic resonance imaging (MRI) [9,10]. For brain imaging, NIR is used to assess brain activities [11,12]. It is now considered a potential alternative to existing brain-imaging modalities such as functional MRI and electroencephalography (EEG), since it is more compact and cheaper. Research groups have assessed multi-modality applications with functional NIR. Regular combinations with functional NIR include EEG or fMRI to concurrently measure brain activity [13]. NIR also has many potential applications in the brain–computer interface [14,15,16]. Recently, there have been many attempts to utilize NIR technology for the continuous monitoring of blood glucose levels [17,18]. Different approaches and designs have been proposed for non-invasive blood glucose measurement through the skin [19,20,21].

The transmission and reflection modes of NIR setups have been investigated [22]. The NIR reflection mode is preferred for thick and dense tissue, while the transmission mode is better for thin tissue. The reflection mode is most common, since the majority of non-invasive NIR applications involve thick and dense tissue. NIR systems for both brain and breast imaging are examples of the NIR reflection mode [7,23]. Blood glucose NIR sensors are also based on the reflection mode, since the glucose contents are found in the skin dermis layer. In a previous work [24], we proposed dual NIR sensors, wherein two channels were used to measure both the signal of interest and the interfering noise arising from the superficial skin layer. Furthermore, the factors affecting the NIR penetration depth and the suitable source-to-detector separation (SDS) for a range of NIR wavelengths were investigated. Recently, attempts were made by researchers [25] to combine both the NIR transmission and NIR reflection configurations for blood glucose measurements. The design used two reflection-based channels and one transmission-based channel. A deep neural network model was then used to predict the glucose blood contents.

NIR technology relies on illuminating the human tissue with a light source and detecting the escaping penetrating light at a distance. The physiological variations within the tissue are related to the light attenuation (*A*) according to the modified Beer–Lambert law [26]. For the tissue medium, the Beer–Lambert law can be stated as follows:

(1) $$A_{\lambda }=\varepsilon_{\lambda }\times C_{\lambda }\times d\times DPF$$

where *ε* is the molar absorption coefficient, *C* is the concentration of the absorbing particles, and *d* is the source-to-detector separation. Depending on the optical properties of the different layers of the tissue and the operating wavelength *λ* of the light source, photons can experience multiple scattering events. Thus, the actual optical pathlength is much larger than source-to-detector separation (optode spacing), and so the differential pathlength factor (DPF) was incorporated into the above equation to relate the actual optical pathlength to the source-to-detector separation [27,28].

Many studies have estimated the optical pathlength and the DPF computationally or experimentally [27,28,29,30]. Studies have shown that the DPF can be estimated directly from direct time-of-flight measurements [27]. Phase-resolved optical spectroscopy has also been utilized to estimate the DPF [29,31]. Computationally, the Monte Carlo method was utilized to estimate the DPF in reflectance photoplethysmography [28] and blood volume and oxygen saturation [30] for two wavelengths. Moreover, researchers have investigated the DPF’s dependence on optode spacing, operating wavelength, and age [32,33]. These studies showed that the DPF depends on the source wavelength and the optical properties. Thus, different tissues have different DPFs at different wavelengths. The optical pathlength increases as the optode spacing (the source-to-detector distance) increases. The results show that if the optode spacing is greater than 2.5 cm, the DPF can be assumed to be constant [28]. Knowing the DPF is of high importance in the quantification of physiological variations within tissue, such as changes in the oxygen saturation in the blood. The significance of the accurate determination of the DPF has been reported for non-invasive near-infrared applications [34,35].

The aim of this study was to systematically investigate the DPF for human skin. More specifically, this work investigated the DPF’s relationship to different wavelengths, source-to-detectors distances, and skin layers. Additionally, we assessed the effect of different skin colors on the DPF values. The Monte Carlo (MC) method was utilized to mimic the different layers of human skin. The adopted MC model allowed us to consider the anatomical variations between skin layers, such as the blood and water volume fractions, melanin concentrations, and optical properties of the skin layers. This work could help improve the accuracy of near-infrared techniques in the measurement of physiological variations within tissue, such as blood glucose levels.

## 2. Methods

### 2.1. Human Skin Model

**Figure 1:**
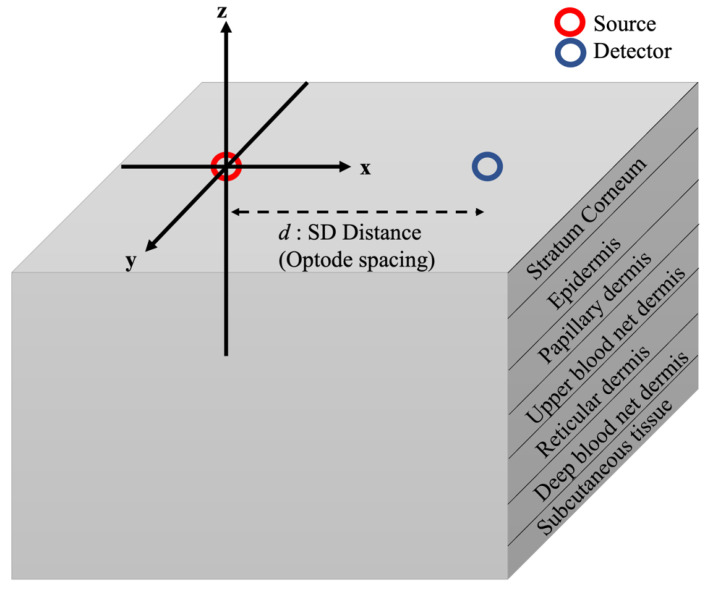
Illustration of the Monte Carlo model for human skin adopted in this study. The light source was placed at the center (0,0,0) of the model, and the detector was placed at a distance (*d*,0,0) away from the source.

The MC skin model adopted in this study is illustrated in Figure 1. The model comprised a semi-infinite slab of seven skin layers. Each skin layer had different optical properties, including the absorption coefficient *μ_a_*, scattering coefficient *μ_s_*, and anisotropy factor *g*. In the three-dimensional cartesian coordinate system presented in Figure 1, the light source was placed at the center (0,0,0) of the model, while the detector was placed at a distance (*d*,0,0) away from the source. The simulation was run for a range of distances (*d*; 0.5 mm to 8 mm, with a step size of 0.5 mm). The maximum distance considered here (*d* = 8 mm) was enough for the NIR to reach the deep blood net dermis of the skin with an acceptable signal-to-noise ratio (SNR) [24]. In particular, the SNR for the shorter wavelengths (550 nm and 650 nm) dropped very quickly as the source-to-detector distance increased. Longer wavelengths (750 nm to 1050 nm) provided better SNRs at longer source-to-detector distances, which meant that deeper tissue layers could be measured [24]. For each distance (*d*), simulations were also conducted for a range of light-source wavelengths. The wavelength range in this model represented the so-called “diagnostic window” of the light spectrum, 450 nm to 1050 nm, with a step size of 100 nm. This window of the spectrum is characterized by its low water-absorption coefficient and, since water is the main absorber in the body, it allows the light to penetrate deeper into the tissue [12,36]. Moreover, the light in this spectrum range is usually utilized to measure oxygenated and deoxygenated blood, since their absorption coefficients are higher than those of water.

This skin model consisted of several layers. The optical properties and the thickness of each layer used to run the simulations are summarized in Table 1. The absorption coefficients of each layer were calculated as explained in [24]. In brief, the blood and water volume fractions *V_blood_* and *V*_H_2_O_ and the absorption coefficients for water, deoxyhemoglobin, and oxyhemoglobin for each layer were used to estimate the layer absorption coefficients. The epidermis layer, which is the skin’s outermost superficial layer, contains melanin, which is responsible for the color of the skin. The absorption coefficient of the melanin in the epidermis was estimated as follows [26,27]:

(2) $$\mu_{a}^{mel}\left(\lambda \right)=6.6\times 10^{10}\times \lambda^{-3.33}$$

Thus, the absorption coefficient for the epidermis was evaluated as:

(3) $$\mu_{a}^{epidermis}\left(\lambda \right)=V_{mel}\;\mu_{a}^{mel}\left(\lambda \right)+V_{H_{2}O}\;\mu_{a}^{HbO_{2}}\left(\lambda \right)+\left(1-\left(V_{mel}+V_{H_{2}O}\;\right)\;\right)\;\mu_{a}^{other}\left(\lambda \right)$$

In this study, the melanin volume fraction $V_{mel}$ was assumed to be 2% and 20%, representing people of light- and dark-colored skin, respectively.

### 2.2. Monte Carlo Simulation

The method of MC simulation is detailed in [31,32]. The light source is modeled as a pencil source directed in the z-direction (Figure 1). Light photons propagate in the tissue, and the reflected (escaped) photons are detected by a detector, which is placed on the same side of the source. In principle, the light photons diffuse in the tissue, following a “banana-shaped” path between the source and the detector [6,33]. Thus, the longer the source-to-detector separation, the deeper the photons can penetrate into the tissue and the higher the photons’ propagation pathlength. In an MC simulation, millions of photons are launched (here: $100\times 10^{6}$ photons) at the start of each simulation run. Each photon packet at its initial launch is given a unity weight (w=1). The weight and the propagation of each photon is calculated based on the optical properties of the tissue. Between each successive scattering event, the photon free pathlength $\left(l\right)$ probability distribution was calculated as:

(4) $$p\left(l\right)=\mu_{s}e^{-\mu_{s}.l}\;$$

During scattering, the direction of the photon is determined by two randomly generated angles, $\left(\emptyset \right)$ and $\left(\varphi \right)$, expressed as:

(5) $$cos\;\emptyset =\frac{1}{2g}\left[1+g^{2}-\left(\frac{1-g^{2}}{1-g+2g\xi }\right)\right]$$

(6) φ=2πξ

After scattering, part of the photon weight is absorbed by the tissue layer, $\Delta w=\frac{\mu_{a}}{\mu_{a}+\mu_{s}}$. If the photon packet has enough energy to reach the boundary of the next tissue layer, it will either be reflected or transmitted. Otherwise, the photon will be terminated. In the case that the photon is transmitted and exits the tissue layer at the detector site (*d*,0,0), the photon is detected. The optical path of the detected photon is also registered [36,37,38,39,40]. The DPF was calculated from the mean optical path $\left(l\right)$ of the detected photon packets at wavelength *λ* for the source–detector separation d as follows:

(7) $$DPF_{\lambda ,d}=\frac{l_{\lambda }}{d}$$

As explained above, this study considered some important parameters. First, we considered the effect of different NIR light-source wavelengths (450 to 1050 nm) on the calculation of the DPF. We also considered a range of source–detector separations (*d* = 0.5 to 8 mm). In addition, the effect of different skin colors (different melanin concentrations) on the DPF was studied. The melanin concentrations considered in this study were 2% and 20%, representing people of light- and dark-colored skin, respectively. In each scenario, the simulations were repeated (N = 20) by independently seeded MC simulations [40,41,42,43,44].

## 3. Results and Discussion

**Figure 2:**
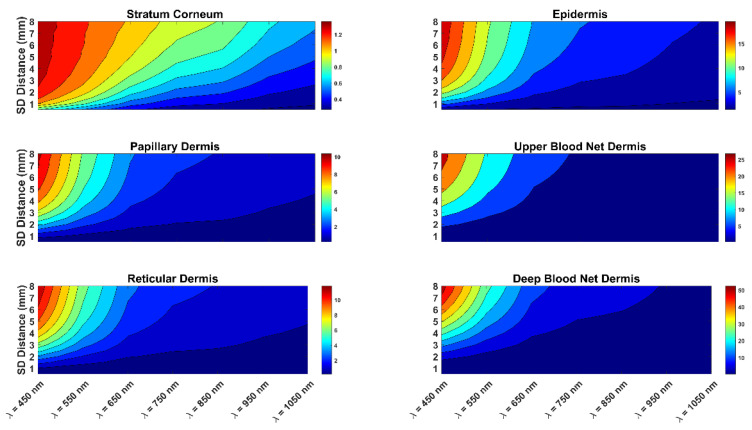
Optical pathlength of light photons for different skin layers at 2% melanin concentration. The figure shows the effects of different light source wavelengths and source-to-detector separations on the optical pathlength of photons.

The optical pathlength of the photons for different skin layers with a melanin concentration of 2% are presented in Figure 2. This figure shows the optical pathlength for different wavelengths and skin layers. The figure also indicates the effect of the source-to-detector separation on the optical pathlength.

**Figure 3:**
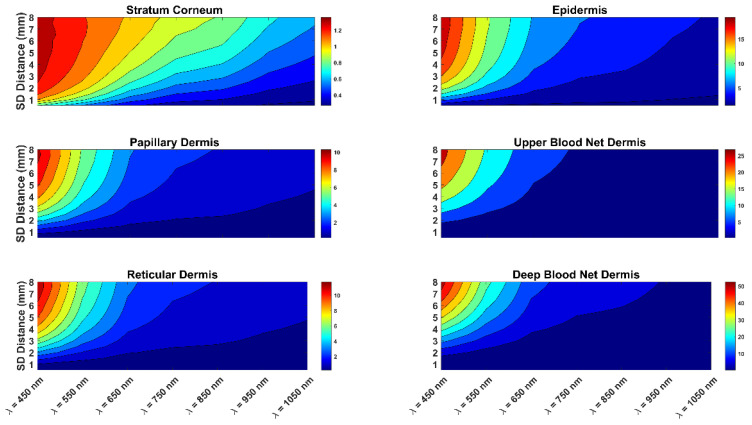
Optical pathlength of the light photons for different skin layers at 20% melanin concentration. The figure shows the effects of different light source wavelengths and source-to-detector separations on the optical pathlength of photons.

In addition, Figure 3 present the optical pathlength of the photons for skin with a 20% melanin concentration. The two figures are similar, showing that the impact of skin color on optical pathlength was not significant. The figures also show that as the wavelength increased from 450 nm to 1050 nm, the optical pathlength clearly decreased. In addition, as the source-to-detector separation increased, there was a clear increase in the optical pathlength. The combination of a short wavelength and a long source-to-detector separation provided the highest optical pathlength values. Similarly, the combination of a longer wavelength and a short source-to-detector separation presented the lowest values of the photon optical pathlength. Thus, in estimating the DPF, it is important to consider the effect of both the wavelength and the source-to-detector separation. It is also important to consider the impact of the wavelength and source-to-detector separation on the DPF when designing an NIR sensor. More specifically, to penetrate deeper tissue layers, one would opt for a longer wavelength.

In addition, the two figures show the details of the optical pathlength of photon propagation in each layer of the skin. In all skin layers, the shorter the wavelength, the higher the photon optical pathlength values. Clearly, the optical pathlength had higher values in the deeper layers of the skin. In comparing the different skin layers (Figure 2 and Figure 3), it is worth noting that the layers most affected were the stratum corneum and the epidermis of the skin. Thus, the effect of the skin color on the optical pathlength was not significant, since the epidermis is very thin in comparison to the other layers.

**Figure 4:**
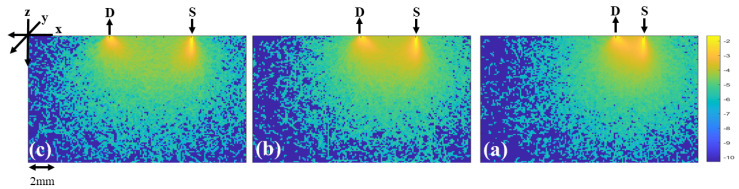
Spatial sensitivity profile of the photon distributions for three different source-to-detector separations ((**a**) 2 mm, (**b**) 4 mm, and (**c**) 6 mm) for the wavelength 1050 nm. Photons follow a so-called “banana-shaped” path, which showed that the optical pathlength increased when the source-to-detector separation increased.

The optical pathlength of photons was larger than the source-to-detector separation. The larger the source-to-detector separation, the higher the penetration depth in the tissue. Figure 4 shows the spatial sensitivity profile of the photon distributions for three different source-to-detector separations: 2 mm, 4 mm, and 6 mm. This figure illustrates that the source-to-detector range used in this study was sufficient to penetrate into the deep blood net dermis, which is a necessary layer for blood content measurement, including glucose. The source-to-detector range verses the depth of penetration of different skin layers is discussed in [24].

**Figure 5:**
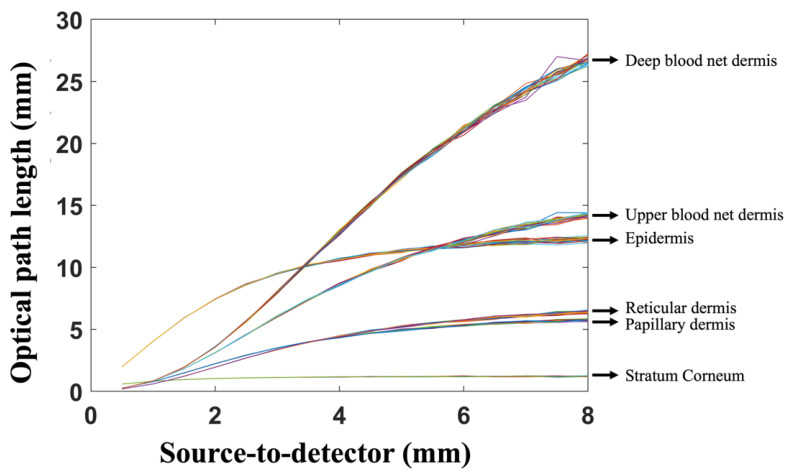
Optical pathlength for different layers at the wavelength of 550 nm. The MC simulations were run independently seeded for N = 20. The number of launched photons in the MC simulations was $100\times 10^{6}$.

Figure 5 shows how the optical pathlength changed for different source-to-detector separations. The figure also shows the number of trials run (N = 20) for each simulation. The number of trials refers to the number repetitions of independently seeded MC simulations. As expected, the standard deviation was very small for the short source-to-detector separations and became larger as the source-to-detector separation increased. Nevertheless, it is clear from the figure that the standard deviation was good enough to clearly differentiate between the different skin layers. Besides the source-to-detector separation, the standard deviation in Figure 5 was also affected by the number of photons launched in the MC simulations. For a lower number of launched photons, the standard deviation tended to be higher for all distances. A high number of launched photons provided more accurate simulations with a low standard deviation. This study included different numbers of launched photons: $10\times 10^{5},\;10\times 10^{6},\;\mathrm{and}\;100\times 10^{6}$ (the results are not shown here). Launching $100\times 10^{6}$ photons provided a low enough standard deviation for all wavelengths and source-to-detector separations (as shown in Figure 5).

Table 2 summarizes the estimated DPF values calculated based on Equation (7). The table shows the DPFs for the different investigated wavelengths. The wavelength was a significant factor when estimating the DPF values. Different wavelengths had different DPFs at different source-to-detector separations. As the source-to-detector separation increased, the DPF increased for short wavelengths (450 nm and 550 nm). For longer wavelengths, it was found that increasing the source-to-detector separation decreased the DPF values.

## 4. Conclusions

In conclusion, this study aimed to investigate the DPF for human skin for a range of source-to-detector separations and wavelengths. The study considered the NIR reflection configuration, since it is the most suited for the majority of non-invasive NIR applications, which are designed for thick and dense tissue. Light photons penetrating the skin can undergo multiple scattering events, and the actual optical pathlength becomes larger than the source-to-detector separation in the reflection-mode configuration. Thus, the DPF must be incorporated in the modified Beer–Lambert law. Several important factors were considered, such as the wavelength, the source-to-detector separation, and the skin color. The wavelength range in this model represented the so-called “diagnostic window” of the light spectrum. The MC simulation method was utilized to mimic the different layers of human skin. The adopted MC model allowed us to consider anatomical variations between the skin layers, such as the blood and water volume fractions, melanin concentration, and optical properties of the skin layers.

It was found that the wavelength and the source-to-detector separations were the most important factors affecting the optical pathlength. Shorter wavelengths had the strongest effect on DPFs when changing the source-to-detector separations. It was also found that the color of the skin was not a significant factor effecting the optical pathlength. Table 2 summarizes the estimated DPF values for the different source-to-detector separations and light-source wavelengths. These values could be used and incorporated into the modified Beer–Lambert law according to the configuration of the NIR sensor. The accurate estimation of the DPF values will lead to the accurate quantification of physiological variations within the tissue. This work could guide researchers in designing an NIR sensor for human skin by providing a better understanding of the actual photon pathlengths in the skin.

## Figures and Tables

**Table 1:** Values used in the Monte Carlo skin model based on [28,29,30] for different skin layers.

Skin Layer	Thickness (mm)	Scattering Coefficient (1/mm)							*V_blood_*	*V*_H_2_O_
		$\mu_{s,450nm}$	$\mu_{s,550nm}$	$\mu_{s,650nm}$	$\mu_{s,750nm}$	$\mu_{s,850nm}$	$\mu_{s,950nm}$	$\mu_{s,1050nm}$		
Stratum Corneum	0.02	60.61	41.74	22.33	15.05	13.32	9.5	7.52	0	0.05
Epidermis	0.25								0	0.2
Papillary dermis	0.1								0.04	0.5
Upper blood net dermis	0.08								0.3	0.6
Reticular dermis	0.2								0.04	0.7
Deep blood net dermis	0.3								0.1	0.7
Subcutaneous tissue	2								0.05	0.7

**Table 2:** Estimated DPF based on Equation (7) for different light source wavelengths and source-to-detector separations.

SDS (mm)	$DPF_{450nm}$	$DPF_{550nm}$	$DPF_{650nm}$	$DPF_{750nm}$	$DPF_{850nm}$	$DPF_{950nm}$	$DPF_{1050nm}$
0.5	9.327229	7.613623	7.734551	8.445864	8.731699	9.321385	9.629079
1	14.26636	9.017028	6.849151	6.742956	6.827569	7.24332	7.628147
1.5	18.92219	10.79773	6.926007	6.171503	6.097595	6.192436	6.473498
2	23.20217	12.52319	7.222662	5.991458	5.791009	5.613065	5.772583
2.5	26.85853	14.18709	7.579191	5.985923	5.645795	5.265959	5.288481
3	30.09627	15.64019	7.971814	6.028839	5.61251	5.041451	4.947292
3.5	32.83371	16.98709	8.331424	6.102681	5.596167	4.896995	4.705665
4	35.20655	18.15731	8.667086	6.193406	5.623161	4.793538	4.519836
4.5	37.30747	19.13173	9.021267	6.297507	5.662255	4.73143	4.384798
5	39.06324	20.0042	9.277562	6.376903	5.692662	4.65514	4.272771
5.5	40.29488	20.77222	9.541198	6.439553	5.731603	4.625515	4.171025
6	41.39538	21.38013	9.748879	6.525236	5.773241	4.581958	4.096541
6.5	42.31183	22.13103	9.885177	6.558909	5.777782	4.535969	4.004704
7	43.00748	22.39849	10.01952	6.623501	5.797535	4.50906	3.953183
7.5	43.06239	22.64816	10.16962	6.62868	5.828102	4.474714	3.897391
8	43.21896	22.80911	10.2432	6.636324	5.813229	4.445111	3.844256

## Data Availability

The data presented in this study are openly available in FigShare at https://doi.org/10.6084/m9.figshare.21895716.v1.
